# Extracellular vesicles derived from hypoxia-preconditioned olfactory mucosa mesenchymal stem cells enhance angiogenesis via miR-612

**DOI:** 10.1186/s12951-021-01126-6

**Published:** 2021-11-21

**Authors:** Lite Ge, Chengfeng Xun, Wenshui Li, Shengyu Jin, Zuo Liu, Yi Zhuo, Da Duan, Zhiping Hu, Ping Chen, Ming Lu

**Affiliations:** 1 Department of Neurology, Second Xiangya Hospital, Central South University, Changsha, 410011 People’s Republic of China; 2 The National & Local Joint Engineering Laboratory of Animal Peptide Drug Development, College of Life Sciences, Hunan Normal University, Changsha, 410081 People’s Republic of China; 3 Hunan Provincical Key Laboratory of Neurorestoratology, The Second Affiliated Hospital, Hunan Normal University, Changsha, 410003 People’s Republic of China; 4 Department of Neurosurgery, The Second Affiliated Hospital of Hunan Normal University, Changsha, 410003 People’s Republic of China

**Keywords:** Olfactory mucosa, Mesenchymal stem cell, Angiogenesis, microRNA, EVs

## Abstract

Mesenchymal stem cells (MSCs) play important roles in tissue repair and regeneration, such as the induction of angiogenesis, particularly under hypoxic conditions. However, the molecular mechanisms underlying hypoxic MSC activation remain largely unknown. MSC-derived extracellular vesicles (EVs) are vital mediators of cell-to-cell communication and can be directly utilized as therapeutic agents for tissue repair and regeneration. Here, we explored the effects of EVs from human hypoxic olfactory mucosa MSCs (OM-MSCs) on angiogenesis and its underlying mechanism. EVs were isolated from normoxic (N) OM-MSCs (N-EVs) and hypoxic (H) OM-MSCs (H-EVs) using differential centrifugation and identified by transmission electron microscopy and flow cytometry. In vitro and in vivo, both types of OM-MSC-EVs promoted the proliferation, migration, and angiogenic activities of human brain microvascular endothelial cells (HBMECs). In addition, angiogenesis-stimulatory activity in the H-EV group was significantly enhanced compared to the N-EV group. MicroRNA profiling revealed a higher abundance of miR-612 in H-EVs than in N-EVs, while miR-612 inactivation abolished the N-EV treatment benefit. To explore the roles of miR-612, overexpression and knock-down experiments were performed using a mimic and inhibitor or agomir and antagomir of miR-612. The miR-612 target genes were confirmed using the luciferase reporter assay. Gain- and loss-of-function studies allowed the validation of miR-612 (enriched in hypoxic OM-MSC-EVs) as a functional messenger that stimulates angiogenesis and represses the expression of *TP53* by targeting its 3′-untranslated region. Further functional assays showed that hypoxic OM-MSC-EVs promote paracrine Hypoxia-inducible factor 1-alpha (HIF-1α)-Vascular endothelial growth factor (VEGF) signaling in HBMECs via the exosomal miR-612-TP53-HIF-1α-VEGF axis. These findings suggest that hypoxic OM-MSC-EVs may represent a promising strategy for ischemic disease by promoting angiogenesis via miR-612 transfer.

**Supplementary Information:**

The online version contains supplementary material available at 10.1186/s12951-021-01126-6.

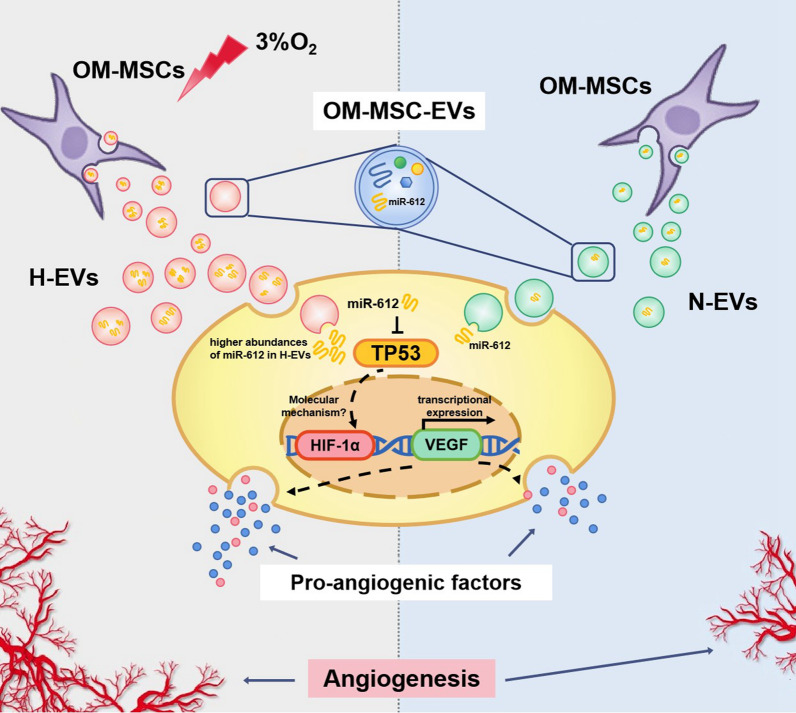

## Introduction

Over the past few years, the mesenchymal stem cell (MSC) therapy has attracted widespread attention for the treatment of ischemic disease, such as skin wound healing, peripheral and coronary vascular disease [1], cerebral infarction [2], and acute kidney ischemia injury [3]. MSCs are tissue-derived cells with unique characteristics that include a self-renewing ability, multilineage differentiation potential, and immunomodulatory properties [4]. Studies in both animal and human settings have demonstrated the therapeutic potential of MSCs in the treatment of a range of disorders, including ischemic disease. Recently, extracellular vesicles (EVs) have been reported to be essential paracrine components of MSCs, and they may offer a suitable alternative to cell-based therapies. Specifically, MSC-derived extracellular vesicles (MSC-EVs) possess an angiogenic function and are highly effective for treating ischemic diseases.

MSC-derived extracellular vesicles (MSC-EVs) may overcome the problems associated with MSC therapy. EVs are small 40–150 nm membrane particles of endosomal origin that play crucial roles in intercellular communication by delivering micro RNAs (miRNAs), mRNAs, and proteins to recipient cells [5, 6]. EVs exhibit stem cell-like pro-regenerative properties and direct treatment with them may avoid many adverse effects of stem cell transplantation therapy [7]. Because EVs are not live cells, the low efficacy of MSC therapy due to poor survival can be overcome by the MSC-EVs therapy. Many studies have reported that the local injection of EVs secreted by human MSCs from different sources can promote angiogenesis, suggesting that EV-based therapy is a promising treatment [8].

The olfactory mucosa (OM) is an attractive source of transplantable stem cells for central nervous system repair, which possesses several distinct advantageous attributes, including lifelong renewal, easy access, no risk to donors, no ethical problems, and autotransplantation potential to avoid immune rejection [9]. It is believed that olfactory mucosa mesenchymal stem cells (OM-MSCs), a novel type of resident stem cells in the olfactory lamina propria, have a high proliferation rate, self-renewal capacity, and ability to differentiate into multiple lineages. In addition to their potential application in tissue repair and regeneration, OM-MSCs have also been utilized as a convenient and effective method for the regeneration of both hippocampal neurons in mice and the spinal cord in humans [10]. Our previous work has identified 274 secreted proteins secreted by OM-MSCs using LC–MS/MS. It is well known that these molecules are important in neurotrophy, angiogenesis, cell growth, differentiation, apoptosis, and inflammation, which are all highly correlated with tissue repair [11]. Moreover, we conducted an in-depth study on the role of olfactory mucosa mesenchymal stem cells (OM-MSCs) in the treatment of ischemic stroke. It was found that OM-MSCs exert neuroprotective effects in cerebral ischemic/reperfusion (I/R) injury via the Golgi apparatus secretory pathway and alleviate mitochondrial dysfunction [12, 13]. However, the direct use of stem cells for therapeutic purposes remains limited by many risk factors, such as tumor formation, thrombosis, poor survival in inflammatory and hypoxic condition, and unwanted immune responses [14, 15]. Additionally OM-MSCs also contain abundant EVs [16]. Nevertheless, few studies to date have directly utilized OM-MSCs to harvest EVs for therapeutic uses.

In recent years, researchers have become committed to using exogenous means to enhance the ability of MSCs to promote angiogenesis and improve ischemia and injury. It has been reported that MSCs cultured in hypoxia (3% O_2_) significantly increases in vitro cell survival, proliferation, and angiogenesis-related growth factors. Furthermore, hypoxia preconditioning of MSCs is a beneficial approach to promote cell survival and treat several diseases, such as spinal cord injury [17] and cerebral ischemia [18]. We have previously demonstrated that hypoxia preconditioning of OM-MSCs can regulate their production of paracrine mediators, conferring neuroprotection against cerebral I/R injury [18]. Therefore, it was hypothesized that the angiogenesis-promoting effect of OM-MSC-EVs might be enhanced by culture in hypoxic conditions. In the present study, we compared pro-angiogenic effect between hypoxia OM-MSC derived EVs (H-EVs) and normoxia-cultured OM-MSC derived EVs (N-EVs) both in vitro and in vivo. Furthermore, advanced studies have shown that miR-612 in OM-MSCs can be transfected into endothelial cells through EVs and promote endothelial cell specification via direct suppression of its target *TP53*. To the best of our knowledge, this is the first study to show that hypoxic OM-MSC-EVs have the ability to promote angiogenesis and to elucidate the underlying mechanism.

## Materials and methods

All experiments were carried out in accordance with the approved guidelines and were approved by Hunan Normal University and the institutional ethical and animal care committees. Human nasal mucosa biopsies were obtained with informed consent and all experiments were approved by the Ethics Committee at the Second Affiliated Hospital of Hunan Normal University (Ethical Approval Document No. 2018-30), and all clinical investigations have been conducted according to the principles expressed in the Declaration of Helsinki.

### Isolation, culture and identification of human OM-MSCs

OM-MSCs were obtained from healthy male volunteers for scientific purposes (20–50 years old) at the Second Affiliated Hospital of Hunan Normal University. The isolation and culture of OM-MSCs were carried out using a protocol from our previous studies. Cell surface markers (CD34, CD45, CD44, CD73, CD90, CD105, CD133, CD146) were used to characterize OM-MSCs by flow cytometric analysis.

### Isolation and identification of normoxia and hypoxia OM-MSC-EVs

Normoxia and hypoxia OM-MSC-EVs were purified by differential centrifugation, as described previously [19]. Before collecting the supernatant, the flow apoptosis of hypoxic and normoxic OM-MSCs was determined, and the supernatant could be collected only if the apoptosis rate was less than 3%. Hypoxia OM-MSCs was cultured at 3% oxygen concentration for 48 h. Briefly, the OM-MSCs were cultured to 90% confluence in complete DMEM; then, the complete medium was replaced with DMEM supplemented with 10% sEV-depleted FBS. The sEV-depleted FBS was prepared by centrifuging FBS at 120,000*g* for 24 h and then passing it through a 0.22-μm filter (Millipore, SLGP033RB). 48 h the conditioned medium was collected and centrifuged at 300 g for 10 min, at 2000*g* for 10 min, and at 10,000*g* for 30 min to remove cells and cell debris. The clarified supernatant was then concentrated with a 0.22 μm syringe filter before EVs preparations. Firstly, the supernatant was then transferred to Ultra-Clear tubes and centrifuged at 100,000*g* for 70 min at 4 °C with a SW32Ti rotor (Beckman Coulter, Netherlands). The exosome-containing pellet was washed with phosphate-buffered saline (PBS) and centrifuged at 100,000*g* for 70 min. Finally, the pellet was then carefully re-suspended in 100 μL PBS. The protein concentration was determined by BCA protein assay kit (Beyotime, China). The samples were used immediately or stored at − 80 °C.

The identification of OM-MSC-EVs were carried out using a protocol from our previous study.The protein content of the concentrated EVs was determined using a bicinchoninic acid (BCA) protein assay kit (Beyotime, China). The OM-MSC-EVs size and concentration was assessed by nanoparticle tracking analysis (NTA) using a Nanosight NS300 (Malvern, UK). EVs markers CD63, CD81 and TSG101 (1:1000, ProteinTech, China) and OM-MSCs marker Nestin (1:1000, ProteinTech, China) were determined using Western blot.

### miRNA arrays

Total RNA extracted from the normoxia and hypoxia OM-MSC-EVs was used for miRNA arrays. miRNA profiling was performed with OE Biotech’s (Shanghai, China) miRNA microarray service based on Affymetrix miRNA 3.0 Array.

### Culture of human brain microvascular endothelial cell and transfection

Human brain microvascular endothelial cells (HBMECs; Cell Bank of the Chinese Academy of Sciences, Shanghai, China) were cultured in Dulbecco’s modified Eagle’s medium: nutrient mixture F12 (DMEM/F12; Invitrogen) with 10% fetal bovine serum (FBS; Invitrogen, United States) at 37℃ in 5% CO_2_ atmosphere. Cells were incubated at 37 °C, 5% CO_2_.

According to the manufacturer’s instructions,when HBMECs at 80% confluency, they were transfected with 50 nM miRNAs using Lipofectamine 2000 in Opti-MEM (Invitrogen). The synthetic miR-612 mimic, miR-612 inhibitor, mimic negative control (NC), inhibitor negative control (NC), were purchased from RiboBio (Guangzhou, China). After transfection for 5 h, the culture medium was replaced with complete medium. Other cells were cultured in 6-well culture plate and transfected with miR-612 inhibitor or the negative control inhibitor using Lipofectamine 2000 (Invitrogen), and cultured in complete medium containing 100 μg/mL normoxia or hypoxia OM-MSC-EVs (200 μg/well) or an equal volume of PBS. After 24 h of incubation, the downstream experiments were performed.

To evaluate the relationship between knocking down or up-regulation of *TP53* expression and miR-612 in endothelial angiogenesis, *TP53* small interfering RNA (siRNAs) (*TP53* siRNA#1, *TP53* siRNA#2, *TP53* siRNA#3) and pcDNA3.1-CMV-*TP53* obtained from RiboBio (Guangzhou, China) were respectively used in HBMECs. Briefly, cells were transfected with si*TP53*, pcDNA3.1-CMV-*TP53* or the universal negative control siRNA (Con siRNA) using Lipofectamine 3000 (Invitrogen) according to the instructions of the manufacturers. 24 h later, the efficiency of these siRNAs and pcDNA3.1-CMV-*TP53* were verified by qRT-PCR. The siRNA sequences used in this study were the following:

- *TP53* siRNA #1: 5′-GTACCACCATCCACTACAA-3′;
- *TP53* siRNA #2: 5′-AGAGAATCTCCGCAAGAAA-3′;
- *TP53* siRNA #3: 5′-GGAGTATTTGGATGACAGA-3′.

### EVs uptake assay

OM-MSC-EVs were labeled with a green fluorescent dye PKH67 (Sigma Alderich, USA) according to the manufacturer’s instructions.Then the cell-labeled suspension was centrifuged at 300*g* for 15 min and the supernatant was discarded. Cells were washed twice with PBS and seeded into culture flasks for 48 h of incubation. Next, EVs were isolated from the conditioned media of MSCs and incubated with HBMECs at 37 °C for 3 h. Cells were then washed with PBS and fixed with 4% paraformaldehyde for 15 min. After washing with PBS, nuclei were stained with DAPI (Invitrogen, USA). The signals were analyzed with a fluorescence microscope (Leica DMI6000B, Germany).

### In vitro on HBMECs

#### Proliferation assay

In brief, cells (5 × 10^3^ cells per well; five replicates per group) were seeded into 96-well culture plates and treated with EVs (100 μg/mL) from different groups or VEGF (30 ng/mL) or PBS. A group without cells served as the blank. On 6 and 12 h, cell counting kit-8 reagent (CCK-8; 10 μL per well; DOJINDO, Japan) was added to the culture medium (100 μL per well). After incubation at 37 °C for 2 h, the absorbance of each well was measured at 450 nm by a microplate reader (Bio-Rad 680, Hercules, USA) and cell proliferation was represented through the mean absorbance of each individual well minus the blank value.

#### Scratch wound healing assay

Cells (5 × 10^5^ cells per well; three replicates per group) were seeded into a 6-well plate and incubated at 37 °C. After the cells had attached, the monolayer was scratched with a p200 pipette tip, washed with PBS to remove floating cells and then exposed to EVs (100 μg/mL) from different groups or an equal volume of PBS. Mitomycin-C (5 µg/mL; Sigma) was present throughout the migration assays to exclude the influence of cell proliferation on wound closure. HBMECs were photographed at 6 h and 12 h after wounding. The rate of migration area was calculated as the ratio of closure area to initial wound as described previously [20]:

$${\text{Migration}}\,{\text{area}}\,{\text{(\% ) = }}{{\left( {{\text{A}}_{0} - {\text{A}}_{{\text{n}}} } \right)} \mathord{\left/ {\vphantom {{\left( {{\text{A}}_{0} - {\text{A}}_{{\text{n}}} } \right)} {{\text{A}}_{0} \times 100,}}} \right. \kern-\nulldelimiterspace} {{\text{A}}_{0} \times 100,}}$$

where A_0_ represents the area of initial wound area and An represents the remaining area of wound at the metering point.

#### Transwell migration assay

Boyden chamber assays were performed using 24-well transwell inserts (Corning, NY, USA) with 8 μm pore-sized filters and 24-well culture plates as described previously [20]. Cells (4 × 10^4^ cells per well; three replicates per group) were suspended in low serum (5% FBS) medium and plated into the upper chamber. 500 μL complete medium (containing 10% FBS) supplemented with EVs (100 μg/mL) from different groups or an equal volume of PBS was added to the lower chamber. After incubation for 16 h, cells attached to the upper surface of the filter membranes were removed by cotton swabs and cells on the bottom side of the filter (the migrated cells) were stained with 0.5% crystal violet for several minutes. The number of migrated cells was quantified under an optical microscope at a 100× magnification (Leica). The absorbance of each well was measured at 550 nm by a microplate reader (Bio-Rad 680, Hercules, USA) and cell migrationn was represented through the mean absorbance of each individual well.

#### Tube formation assay

130 μL cold Matrigel per well was transferred into each well of a 24-well plate and incubated at 37 °C for 30 min. Then, HBMECs (2 × 10^4^ cells per well; three replicates per group) were plated into the Matrigel-coated 24-well plates and treated with EVs (100 μg/mL) from different groups or PBS. 6–8 h after seeding, tube formation was detected under an inverted microscope (Leica). The indicators ( total number of branches length, junctions, nodes and meshes) revealing the abilities to form tubes were measured by using Image-J software.

### In vivo matrigel plug assay

Athymic‐nude (nu/nu) mice (female, 7–8 weeks old) were purchased from Slac Laboratory Animal Co., Ltd. (Shanghai, China) and were used for in vivo Matrigel plug studies. At the end of the experiment mice were euthanized by CO_2_ inhalation. Mice were randomly allocated to 12 groups (n = 6 mice per group for the groups): (1) Matrigel only, (2) Matrigel + N-EVs, (3) Matrigel + H-EVs, (4) Matrigel + HBMECs, (5) Matrigel + HBMECs + N-EVs, (6) Matrigel + HBMECs + H-EVs, (7) Matrigel containing HBMECs transfected with an miR-612 agomir negative control (agomir NC group), (8) miR-612 agomir (miR-612 agomir group), (9) miR-612 antagomir negative control (antagomir NC group), (10) miR-612 antagomir (miR-612 antagomir group), (11) miR-612 antagomir + H-EVs (miR-612 antagomir + H-EVs group), and (12) miR-612 antagomir + N-EVs (miR-612 antagomir + N-EVs group).

To test whether normoxia or hypoxia OM-MSC-EVs affected angiogenesis, normoxia and hypoxia OM-MSC-EVs (100 μg/mL) were resuspended in 500 μL of ice-cold Matrigel respectively, and implanted subcutaneously on the back of nude mice, while an equal volume of Matrigel without EVs was implanted as negative control. Matrigel plugs were removed for analysis 14 days later.

To test whether normoxia or hypoxia OM-MSC-EVs affected HBMECs angiogenesis, 5 × 10^6^ HBMECs were mixed with 500 μL of Matrigel (BD, USA) at a ratio of 1:1, while normoxia and hypoxia OM-MSC-EVs (100 μg/mL) were resuspended in 500 μL of ice-cold Matrigel respectively, and implanted subcutaneously on the back of nude mice, while an equal volume of Matrigel with 5 × 10^6^ HBMECs was implanted as negative control. Matrigel plugs were removed for analysis 14 days later.

To determine whether exosomal miR-612 is responsible for the proangiogenic effect of normoxia and hypoxia OM-MSC-EVs, HBMECs were loaded with miR-612 agomiR NC/miR-612 agomiR (1.5 nmol/mouse) and miR-612 antagomiR NC/miR-612 antagomiR (3 nmol/mouse), respectively. Next, 5 × 10^6^ HBMECs were mixed with 500 μL of Matrigel (BD, USA) at a ratio of 1:1, while an equal volume of Matrigel with normoxia and hypoxia OM-MSC-EVs (100 μg/mL) were implanted as negative control. Then, the cell suspensions were injected subcutaneously in the dorsal region of nude mice. Finally, the Matrigel plugs were removed for analysis on Day 14.

### Histology and immunofluorescence

Matrigel plugs were collected, fixed with 4% PFA, embedded in paraffin, and sectioned. For immunohistochemical analyses, Matrigel plug sections were stained with primary antibodies against CD31 (1:100, ab222783, abcam) and DAPI (Invitrogen, USA) as previously described [21].

### Bioinformatics analysis and luciferase reporter assay

Putative targets of miR-612 were searched using TargetScan (http://targetscan.org/) and miRDB (http://www.mirdb.org/). *TP53*, which was predicted as a target of miR-612, was then assessed by luciferase reporter assay. The 3′-UTR of *TP53* containing either wild-type (WT) or mutant-type (MT) binding sites of miR-612 were synthesized by GenePharma Co., Ltd. and inserted into the pmirGLO vector (Promega), with the resultant constructs denoted as WT-*TP53* and MT-*TP53*, respectively. HEK293 cells were cotransfected with miR-612 mimics or miR-NC and reporter plasmids WT-*TP53* or MT-*TP53* using Lipofectamine 2000. The activities of Renilla and firefly luciferase were examined using a Dual-Luciferase^®^ Reporter assay kit from Promega based on the manufacturer's protocols. The activity of firefly luciferase was normalized to that of Renilla luciferase.

### RNA isolation and quantitative real-time PCR

Total RNA from cells and tissues, and exosome-derived RNA were extracted using TRIZOL (Tiangen, Beijing, China). The primers used in this study were synthesized by Sangon Biotech Company (shanghai, China) (Table 1). Using GAPDH and U6 as internal reference primers, the relative mRNA expression of target genes was calculated by the 2 −△△Ct method [22].

**Table 1:** Primer sequence for qRT-PCR

miRNA/mRNA	Sequence (5′-3′)	
GAPDH	F → ACAGCCTCAAGATCATCAGC	R → GGTCATGAGTCCTTCCACGAT
U6	F → CTCGCTTCGGCAGCACA	R → AACGCTTCACGAATTTGCGT
hsa-miR-612		R → GCTGGGCAGGGCTTCTGAG
TP53	F → ACATTCTCCACTTCTTGTTCCCC	R → CTCCCCACAACAAAACACCAGT
HIF-1α	F → TGGTATTATTCAGCACGACT	R → GCCAGCAAAGTTAAAGCATC
VEGF	F → AGGGCAGAATCATCACGAAGT	R → AGGGTCTCGATTGGATGGCA

### Western blotting

EVs, cells and matrigel tissue were processed for Western blot as described [12, 13]. Immunoblot analyses were performed using the following primary antibodies against Calnexin (1:1000, ProteinTech, China), TSG101 (1:1000, ProteinTech, China), CD81 (1:1000, ProteinTech, China), HIF-1α (5 µg/mL, abcam, UK), VEGF (5 µg/mL, abcam, UK), TP53(1:1200, ProteinTech, China), GAPDH (1:3000, ProteinTech, China).The anti-rabbit IgG and anti-mouse IgG secondary antibodies were obtained from Proteintech. The proteins were visualized using an enhanced chemiluminescent (ECL) detection kit (Advansta Inc., United States).

### Statistical analysis

All experiments were performed in at least three replicates. Data are expressed as mean ± SEM. Differences between groups were estimated using two-side dunpaired Student’s *t*-test or two-sided ANOVA with the Bonferroni correction for thepost hoc *t*-test as appropriate. Statistical analysis was conducted with GraphPad Prism 6 Software (La Jolla, CA, United States). Differences with the probability of P < 0.05 were considered significant.

## Results

### Identification of normoxic and hypoxic OM-MSCs and OM-MSC-EVs

To study the roles of EVs in endothelial cell angiogenesis, normoxic and hypoxic OM-MSCs were first isolated and characterized as previously described [23]. Flow cytometry analysis revealed that both normoxic and hypoxic OM-MSCs were highly positive for MSC surface markers, including CD44, CD73, CD90, CD105, CD133, CD146, and CD29, but negative for CD34 and CD45 (Additional file 1: Fig. S1A). All of these results were consistent with the findings of previous studies [23].

**Fig. 1:**
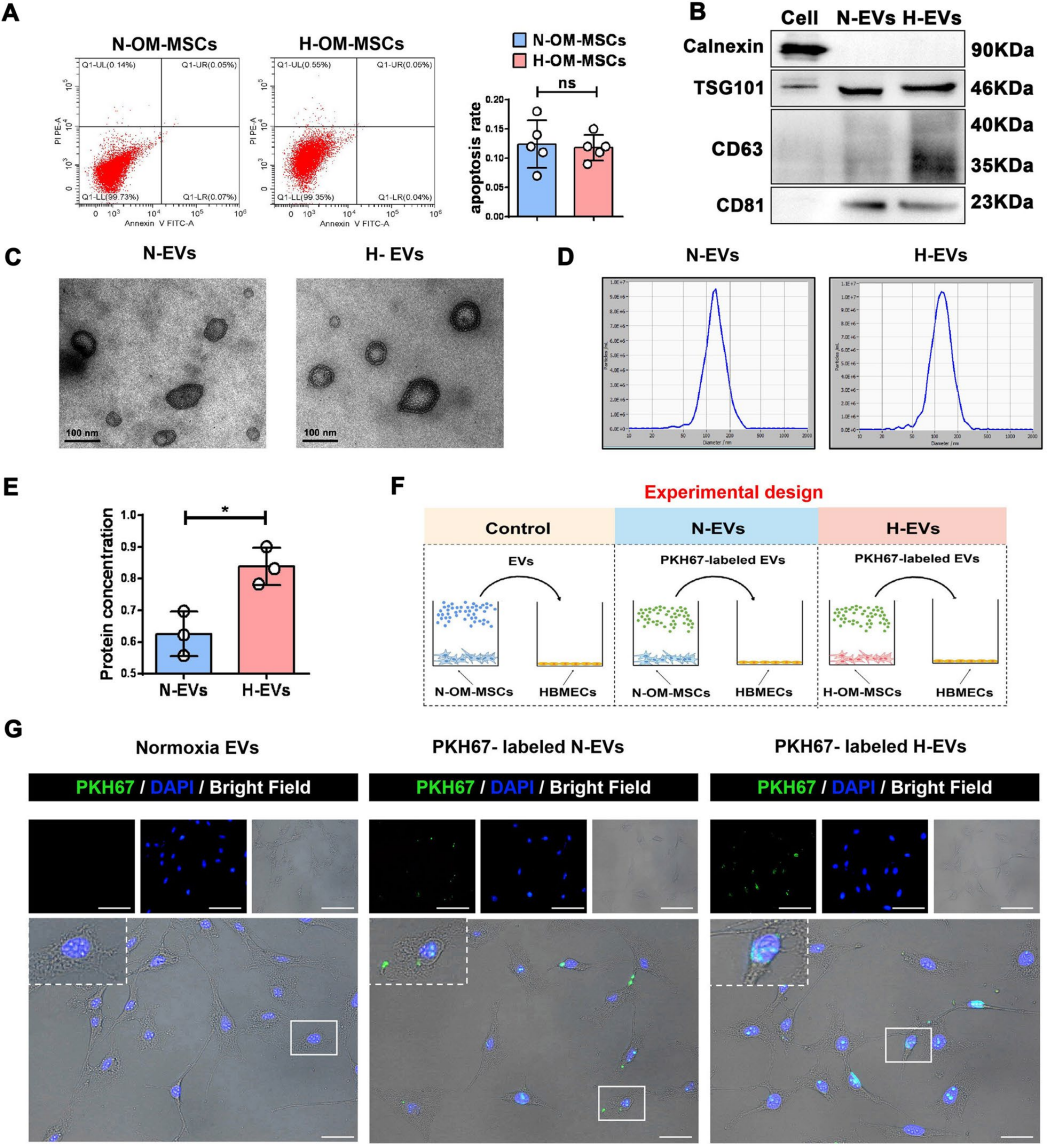
Identification of N-EVs and H-EVs. **A** Flow cytometric analysis showed the levels of apoptosis in N-OM-MSCs and H-OM-MSCs. Both apoptosis rates are less than 0.5% and there are no significant difference. (n = 5). (ns: no significance). **B** Western blot detected the expression of positive markers (TSG101, CD63, nad CD81) and negative markers (Calnexin) in H-EVs and N-EVs. **C** Morphological characteristic of the H-EVs and N-EVs were analyzed using TEM images, Bar = 100 nm. **D** Size distribution analysis of the H-EVs and N-EVs, analyzed by nanoparticle tracking assay (NTA). **E** The BCA assay quantified the protein concentration of H-EVs and N-EVs secreted by equal cells. (n = 3).*P < 0.05. **F**, **G** The schematic representation of EVs uptake experimental design and fluorescence image of PKH67-labeled H-EVs and N-EVs uptake by HBMECs, Bar: upper, 200 μm; lower, 50 μm.

In accordance with the requirements of minimal information for studies of extracellular vesicles 2018 (MISEV 2018) [24], hypoxic and normoxic OM-MSC apoptosis rates were analyzed by flow cytometry before extracting the supernatant. The apoptosis rates of hypoxic OM-MSCs (0.124 ± 0.018, n = 5) and normoxic OM-MSCs (0.118 ± 0.010, n = 5) were < 0.5% to eliminate the influence of apoptosis on the acquisition of EVs (Fig. 1A). H-EVs and N-EVs were isolated from hypoxic and normoxic OM-MSCs and characterized using western blotting, electron microscopy, and NTA assays. Western blotting demonstrated that the exosomal marker proteins TSG101, CD63, and CD81 were present in these EVs as expected, and negative exosome marker calnexin was not detected in both OM-MSC-derived EV types (Fig. 1B). Transmission electron microscopy analysis showed that both types of EVs were round-shaped, with a diameter range of 30–100 nm (Fig. 1C). NTA results revealed a size distribution range of 30–150 nm for normoxic and hypoxic OM-MSC-EVs (Fig. 1D). Bicinchoninic acid protein assay was used to quantify the EV protein concentration as previously described [25]. The total protein amount was 0.620 ± 0.070 μg/μL for N-EVs and 0.832 ± 0.059 μg/μL for H-EVs (*P* < 0.01, n = 3; Fig. 1E). Therefore, there was a difference between the total protein concentrations in the two types of EVs. Collectively, the exosomal characteristics observed in the present study were consistent with those previously reported [16].

As a type of natural nanoparticle, EVs regulate the function of neighboring and distant cells by delivering diverse factors. To evaluate exosome internalization, HBMECs were incubated with the green fluorescent dye (PKH67)-labelled EVs for 6 h and observed using fluorescence microscopy (Fig. 1F). Green fluorescence was detected in the HBMECs, suggesting the internalization of both labeled H-EVs and N-EVs by HBMECs (Fig. 1F, G). Reassuringly, no fluorescent signal was observed in the cells receiving the dyes alone (Additional file 1: Fig. S1B) or unlabeled MSC-EV controls (Fig. 1F, G), showing that the signal was specific to the MSC-EVs being studied.

### H-EVs enhance the angiogenic activities of HBMECs in vitro

**Fig. 2:**
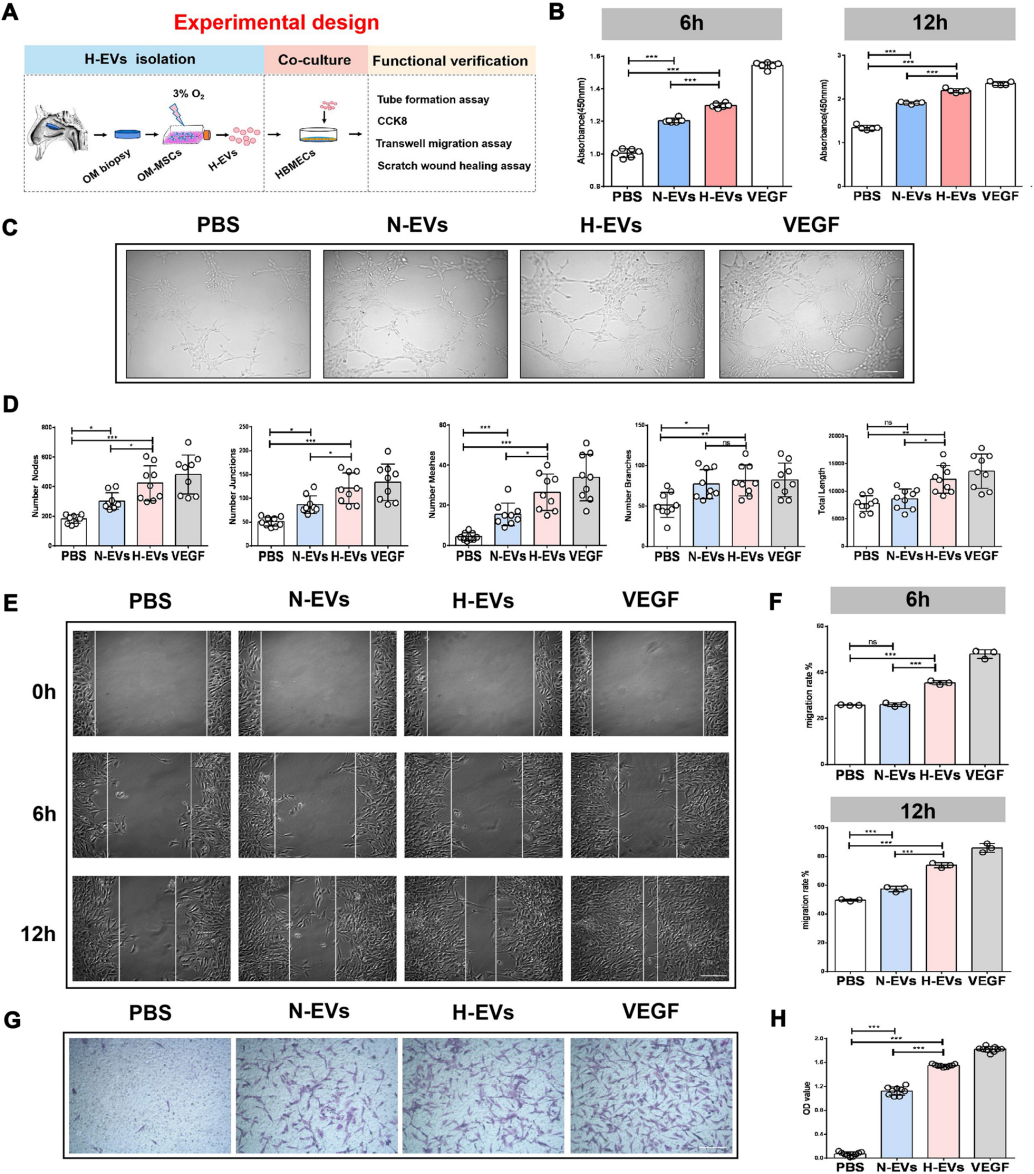
H-EVs enhance the angiogenic activities of HBMECs in vitro*.* A The schematic representation of In vitro experimental design. **B** CCK-8 analysis was applied to measure the effect of H-EVs and N-EVs on proliferation of HBMECs, (n = 6), ***P < 0.001. **C** Representative images of tubule formation for each treatment group, Scale bar: 200 μm. **D** Quantitative data of tube formation using ImageJ, (n = 9), ns: no significance, *P < 0.05, **P < 0.01, ***P < 0.001. **E** Representative images of wound healing assay for each treatment group, Scale bar: 200 μm. **F** Quantitative data of migration rate using ImageJ, (n = 3), ns: no significance, ***P < 0.001. **G** Representative images of transwell assay for each treatment group, Scale bar: 100 μm. **H** Quantitative data of migrating cells OD value using ImageJ. (n = 9), ***P < 0.001

The impact of H-EVs on the angiogenic activities of endothelial cells was then assessed (Fig. 2A). CCK-8 analysis was used to measure the effect of normoxic and hypoxic OM-MSC-EVs on the proliferation of HBMECs. Both N-EVs and H-EVs exhibit similar properties to VEGF (a positive control) and are able to stimulate the proliferation of HBMECs (Fig. 2B). In addition, compared to N-EVs, H-EVs can better promote the proliferation of HBMECs. The tube formation assay on Matrigel was used as an in vitro model of angiogenesis. HBMECs treated with H-EVs and N-EVs showed a higher number of capillary-like structures compared to the control group (Fig. 2C). Quantitative measurements revealed that the branch length, junctions, nodes, meshes, length, and branch number were all significantly increased after normoxic and hypoxic OM-MSC-EV stimulation. Moreover, compared with N-EVs, HBMEC tube formation was significantly enhanced in the H-EV group as determined by the increase in the branch length and total number of junctions, nodes, and meshes (Fig. 2D). Similar results were found in scratch wound healing assay (Fig. 2E, F) and transwell assay (Fig. 2G, H). These findings indicate that OM-MSC-EVs augment the angiogenic activities of endothelial cells, and H-EVs significantly promote angiogenesis compared with the normoxic group.

### miRNA expression profile of H-EVs

**Fig. 3:**
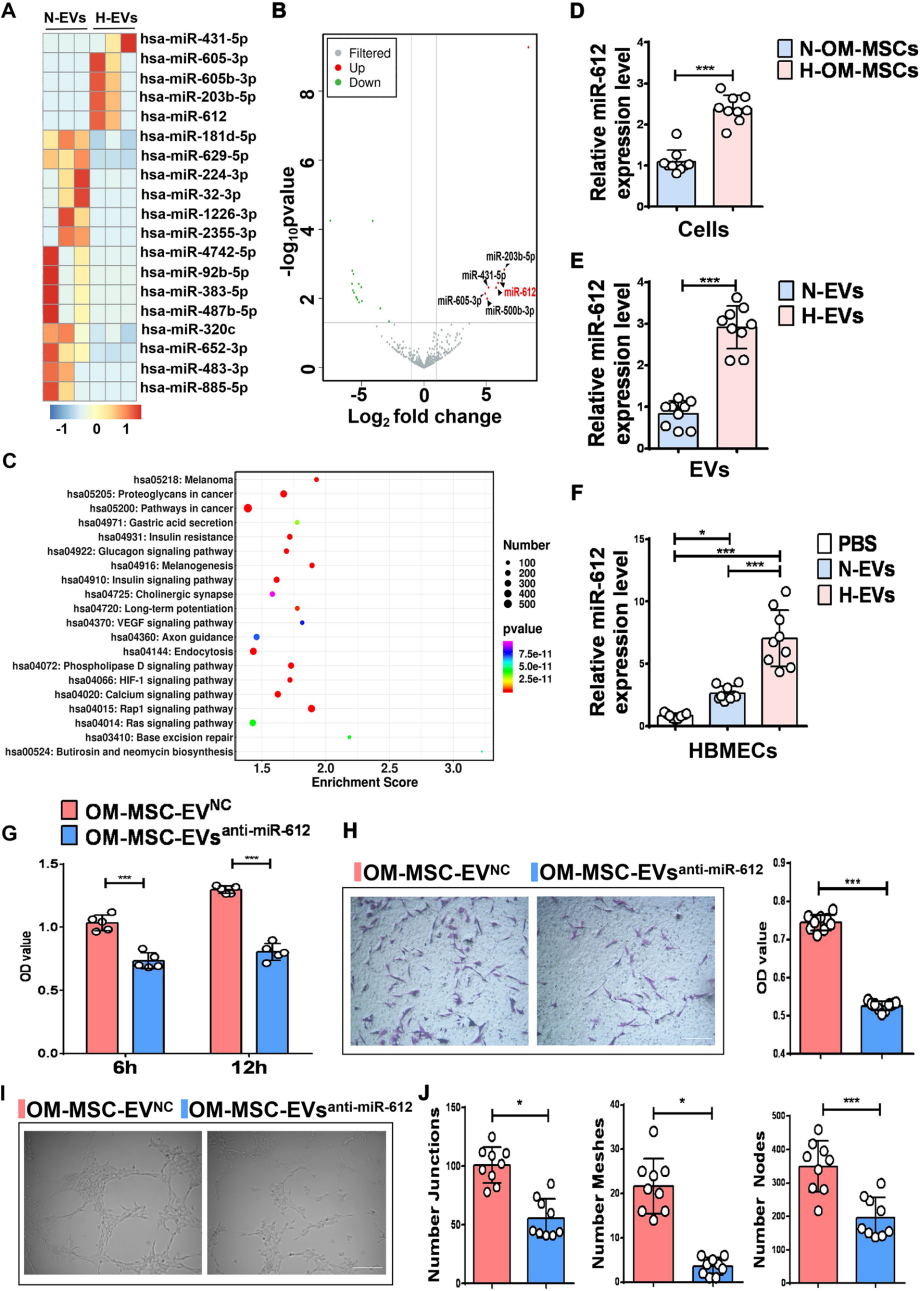
Hypoxia Leads to Changes in H-EVs and N-EVs miRNA Profiles and miR-612 mediates the pro-angiogenic effects of OM-MSC-EVs on HBMECs. **A** Differential expression level of exosomal miRNAs between H-EVs and N-EVs. MiR-612 is one of the exosomal miRNAs with markably greater abundance in H-EVs compared to N-EVs. **B** Volcano plot of differentially expressed miRNAs between H-EVs and N-EVs. Red, significantly upregulated miRNAs in H-EVs; green, significantly downregulated miRNAs in N-EVs; gray, no significant difference. Fold change > 2 and* P* < 0.05 were considered significant. The sequencing samples of H-EVs and N-EVs were isolated from six sample. **C** KEGG pathway analysis of the target genes of five significantly upregulated miRNAs in H-EVs. Top 20 enriched pathways are indicated. **D** Expression level of miR-612 in H-OM-MSCs and N-OM-MSCs, (n = 9), ***P < 0.001. **E** MiR-612 abundance in EVs secreted by H-EVs and N-EVs, (n = 9), ***P < 0.001. **F** After 3 h of H-EVs or N-EVs treatment, the levels of miR-612 in HBMECs were measured by qPCR analysis. Data were normalized to levels of U6 (cellular) or total protein of EVs, (n = 9), *P < 0.05, ***P < 0.001. **G** Cell viability at 6 h and 12 h was examined in HBMECs that were treated with EVs obtained from OM-MSCs that were pretreated with scramble (OM-MSC-EVs^NC^)or with EVs isolated from OM-MSCs that were pretreated with anti-miR-612 (OM-MSC-EVs^anti-miR-612^), (n = 5),***P < 0.001. **H** The migration of HBMECs stimulated by OM-MSC-EVs^NC^ and OM-MSC-EVs^anti-miR-612^ was detected by the transwell assay, (n = 9), ***P < 0.001, Scale bar: 100 μm. **I** Representative images of the tube formation assay in HBMECs treated with OM-MSC-EVs^NC^ and OM-MSC-EVs^anti-miR-612^,Scale bar: 200 μm. **J** Quantitative analyses of the total junctions, meshes and nodes in (I), (n = 9), *P < 0.05, ***P < 0.001

EVs play a vital role in intercellular communication, and their functions mainly depend on their internal contents. Therefore, deep sequencing of small RNAs from H-EVs and N-EVs was conducted. After trimming low-quality reads, contaminants, adaptors, and reads smaller than 17 nt, the remaining reads were mapped to noncoding RNA databases. Additional file 1: Fig. S2A shows the reads identified for categories of small RNA [ribosomal RNA (rRNA), transfer RNA (tRNA), small nuclearRNA (snRNA), cis-regulatory element (Cis-reg), other-Rfam-RNA, gene, repeat, known-miRNA, and unannotation]. The percentage of miRNAs in the total RNA isolated from normoxic and hypoxic EVs corresponded to 22.20% ± 3.10% (n = 3) and 20.75% ± 5.87% (n = 3), respectively. There was no significant difference (*P* > 0.05) between the normoxic and hypoxic conditions (Additional file 1: Fig. S2B). Over 715 and 716 miRNAs were identified in H-EVs and N-EVs, respectively (Additional file 1: Fig. S2C; Additional file 2: Table S1). A total of 286 miRNAs were simultaneously identified both in H-EVs and N-EVs. The number of overlapping and unique proteins between the two groups is shown in Additional file 1: Fig. S2C. A set of miRNAs that were differentially expressed in H-EVs vs. N-EVs was also identified. Among these differentially expressed miRNAs, Fig. 3A shows the 19 miRNAs exhibit the greatest difference in abundance between normoxia and hypoxia in the OM-MSC-EVs (fold change > 2, *P* < 0.05). These include five up-regulated miRNAs and fourteen down-regulated miRNAs. MiR-612 is one such up-regulated miRNA (Fig. 3B). Then, target gene prediction for the upregulated miRNAs, gene ontology (GO; Additional file 1: Fig. S2D), and Kyoto Encyclopedia of Genes and Genomes (KEGG) analyses (Fig. 3C) were performed. Interestingly, hypoxia-inducible factor 1-alpha (HIF-1α) and vascular endothelial growth factor (VEGF) signaling pathways related to angiogenesis were involved in the top 20 KEGG pathways, while miR-612 was involved both in the HIF-1α and VEGF signaling pathways. The results showed that hypoxic OM-MSC intracellular levels of miR-612 were two-fold greater than those in normoxic OM-MSCs (Fig. 3D), which led to increased levels of miR-612 in the EVs (Fig. 3E). To verify the transfection of miR-612 into recipient cells, HBMECs were treated with normoxic and hypoxic OM-MSC-EVs. The cells were then harvested for qRT-PCR analysis. The miR-612 levels in HBMECs were remarkably increased after the cells were stimulated with H-EVs and N-EVs for 3 h (Fig. 3F). Moreover, the miR-612 level was significantly increased in HBMECs treated with H-EVs compared to in the N-EVs treated group (Fig. 3F). These results indicate that miR-612 can be transfected into target cells. It was thus hypothesized that hypoxic OM-MSC-EV-enriched miR-612 might enhance the angiogenic activities of HBMECs.

### Hypoxic OM-MSC-EV-enriched miR-612 enhances the angiogenic activities of HBMECs

To confirm whether miR-612 has a functional role in the EV-induced regulation of angiogenesis, EVs were obtained from OM-MSCs that were pretreated with an anti-miR-612 oligonucleotide (OM-MSC-EVs^anti-miR-612^) or with a scrambled construct as the control (OM-MSC-EV^NC^). The proliferation of HBMECs was quantified using CCK-8 analysis. The OM-MSC-EV^anti-miR-612^ stimulation resulted in a significant decrease in HBMEC proliferation when compared with controls (Fig. 3G). Similar results were also found in the transwell assay (Fig. 3H) and tube formation assay (Fig. 3I, J). The pro-migratory effect of OM-MSC-EVs was attenuated, but not totally abolished in the OM-MSC-EVs^anti-miR-612^ group when compared with the controls (Fig. 3H). Tube formation assay showed fewer capillary-like structures on Matrigel with HBMECs treated with OM-MSC-EVs^anti-miR-612^ compared with the OM-MSC-EVs^NC^ (Fig. 3I). Quantitative analysis of the total junctions, meshes, and nodes further confirmed that down-regulation of miR-612 in OM-MSC-EVs blocked their positive effects on tube formation (Fig. 3J).

**Fig. 4:**
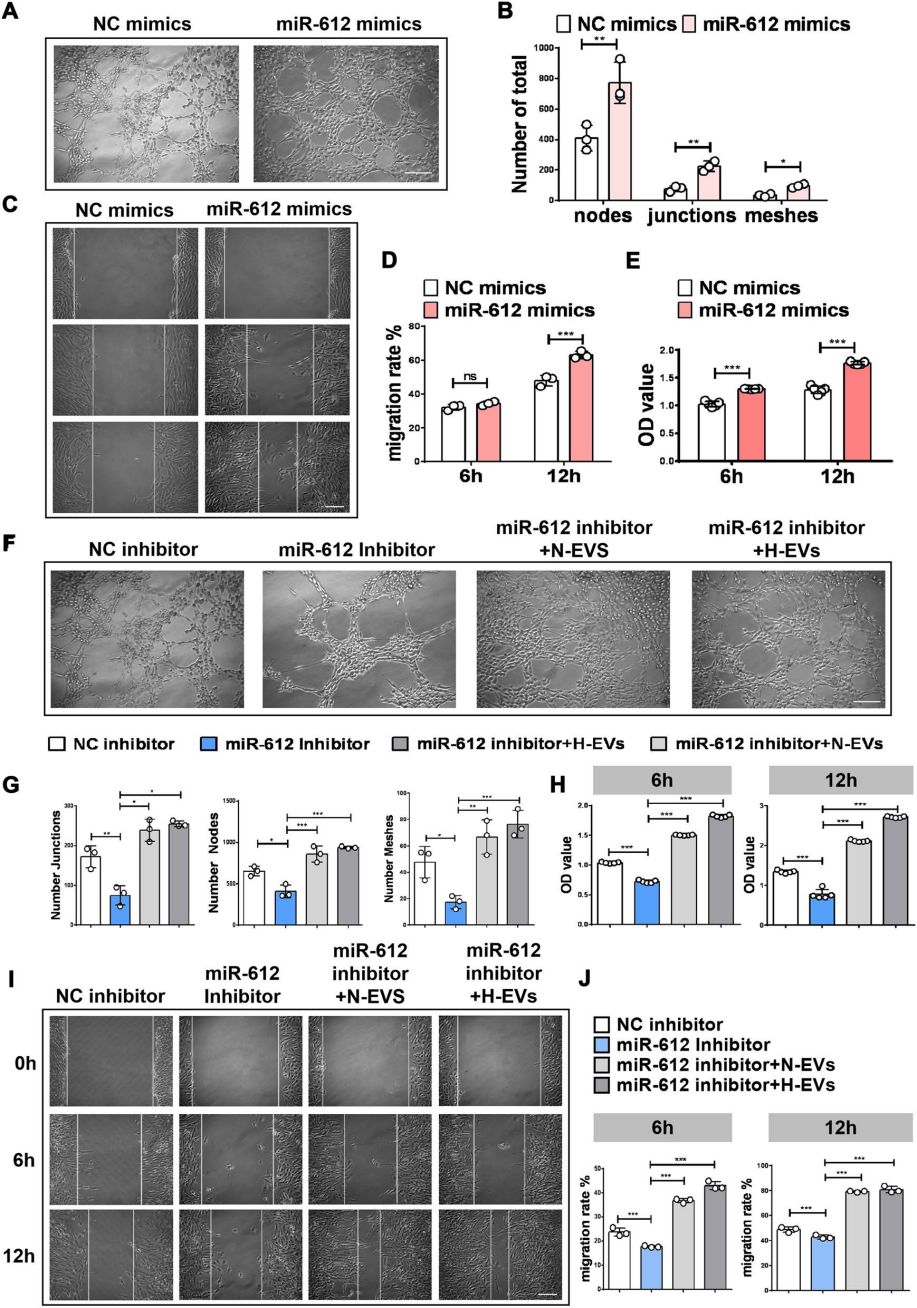
Hypoxic OM-MSC-EV-enriched miR-612 enhances the angiogenic activities of HBMECs. **A** Representative images of tubule formation for NC mimics and mi-612 mimics, Scale bar: 200 μm. **B** Quantitative analyses of the total junctions, nodes and meshes in **A** using ImageJ, (n = 3),*P < 0.05, **P < 0.01. **C** Representative images of wound healing assay for NC mimics and mi-612 mimics, Scale bar: 200 μm. **D** Quantitative data of migration rate using ImageJ, (n = 3), ***P < 0.001, ns: no significance. **E** CCK-8 analysis was applied to measure the effect of NC mimics and mi-612 mimics on proliferation of HBMECs, (n = 3), ***P < 0.001. **F** Representative images of tubule formation for each treatment group, Scale bar: 200 μm. **G** Quantitative data of tube formation using ImageJ, (n = 3),*P < 0.05, **P < 0.01, ***P < 0.001. **H** CCK-8 analysis was applied to measure the effect of each treatment group on proliferation of HBMECs, (n = 5), ***P < 0.001. **I** Representative images of wound healing assay for each treatment group, Scale bar: 200 μm. **J** Quantitative data of m migration rate using ImageJ, (n = 3),***P < 0.001

Thereafter, the effects of miR-612 on the regulation of angiogenesis were further analyzed using miR-612 mimics and its inhibitor in HBMECs. HBMECs transfected with the miR-612 mimics recapitulated the positive effects on tube formation (Fig. 4A, B), migration abilities (Fig. 4C, D), and cell viability (Fig. 4E) when compared with the scrambled group. These assays demonstrated that HBMECs exhibited a much stronger angiogenic ability, proliferative ability, and motility when transfected with miR-612 mimics. These findings indicate that miR-612 augments the angiogenic activities of endothelial cells. Conversely, transfection of HBMECs with the miR-612 inhibitor yielded the opposite results (Fig. 4F–J). The angiogenic ability, proliferative ability, and motility were decreased in response to the treatment with the miR-612 inhibitor (Fig. 4F–J). These results were again reversed when the HBMECs transfected with the inhibitor were incubated with H-EVs and N-EVs (Fig. 4F–J). Collectively, these findings suggest that miR-612 is required for OM-MSC-EV-induced promotion of endothelial angiogenesis.

### OM-MSC-EV-transferred miR-612 modulates HBMEC specification via *TP53*

**Fig. 5:**
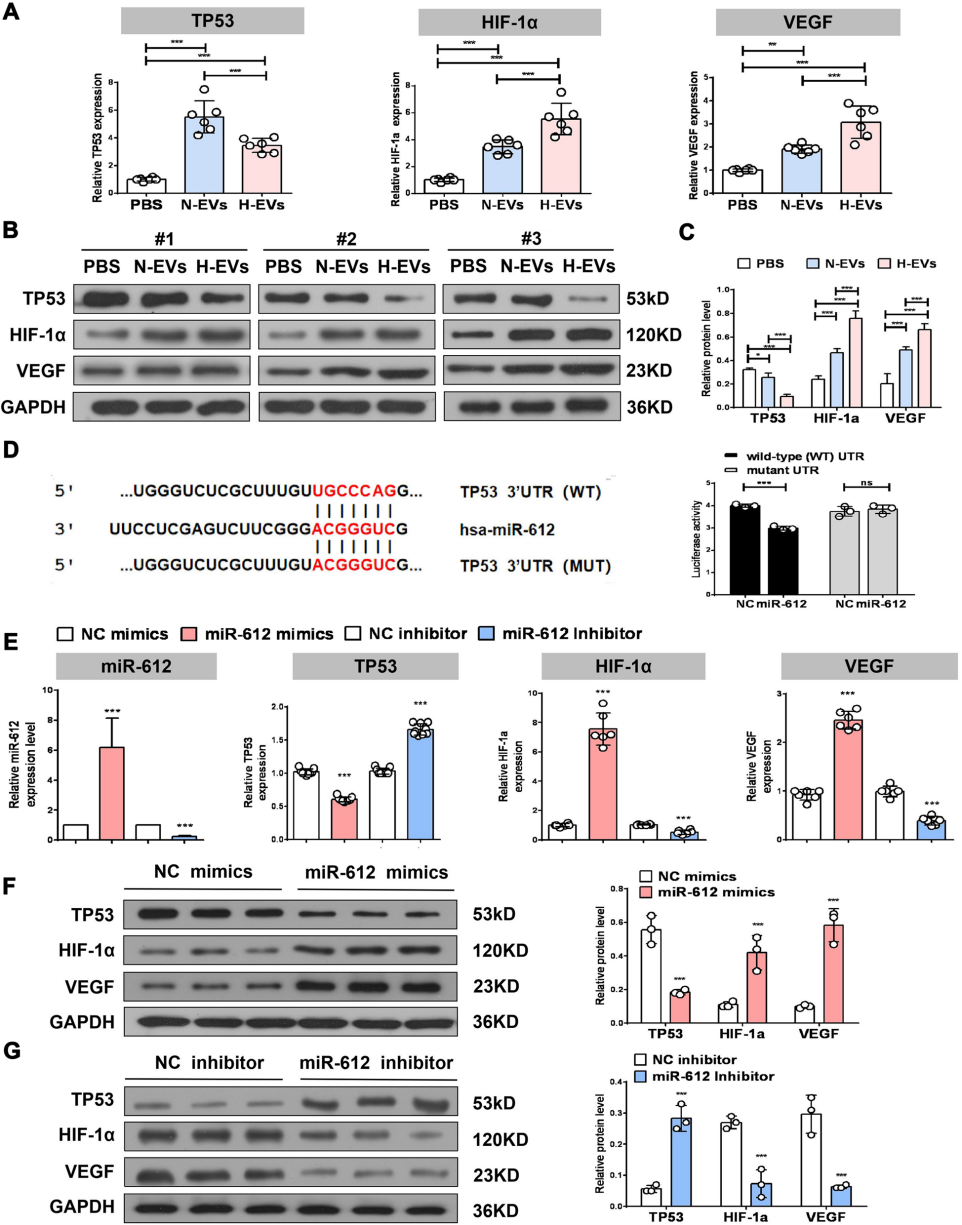
OM-MSC-EVs transferred miR-612 modulates HBMECs specification through TP53. **A** qPCR analysis of the expression of TP53, HIF-1α and VEGF in HBMECs treated with H-EVs and N-EVs, (n = 6), **P < 0.01, ***P < 0.001. **B** Western blot analysis of the expression of TP53, HIF-1α and VEGF in HBMECs treated with H-EVs and N-EVs. **C** Quantitative data of western blot analysis, (n = 3),*P < 0.05, ***P < 0.001. **D** The bioinformatic tool TargetScan to identify putative TP53 was one of predicted target of miR-612.miR-612 mimic significantly decreased the relative luciferase activity of the wild-type vectors, while the luciferase activity of mutant vectors was not altered by miR-612, (n = 3), ***P < 0.001, ns: no significance. **E** qPCR analysis of the expression of miR-612, TP53, HIF-1α and VEGF in HBMECs treated with each treatment group, (n = 6), ***P < 0.001. **F** Western blot analysis of the expression of TP53, HIF-1α and VEGF in HBMECs treated with NC mimics and miR-612 mimics,and quantitative data of western blot analysis, (n = 3), ***P < 0.001. **G** Western blot analysis of the expression of TP53, HIF-1α and VEGF in HBMECs treated with NC inhibitor and miR-612 mimics inhibitor,and quantitative data of western blot analysis, (n = 3), ***P < 0.001

It has been reported that *TP53* inhibition can upregulate HIF-1α and VEGF expression. Interestingly, the levels of *TP53* mRNA and protein were reduced in HBMECs treated with H-EVs and N-EVs, and the decrease in *TP53* in the hypoxic group was greater than that in the normoxic group (Fig. 5A–C). However, the mRNA and protein levels of HIF-1α and VEGF were increased in both groups, while the expression of HIF-1α and VEGF in the hypoxic group was higher than that in the normoxic group (Fig. 5A–C). To investigate the mechanism by which miR-612 promotes angiogenesis, the bioinformatics tool TargetScan was used to identify putative targets of miR-612. *TP53* was one of the predicted targets of miR-612 (Fig. 5D). Therefore, luciferase vectors containing the wild-type or mutant 3′-untranslated region (UTR) sequence of *TP53* were constructed. The miR-612 mimic significantly decreased the relative luciferase activity of the wild-type vectors, while the luciferase activity of mutant vectors was not altered (Fig. 5D). These findings suggest that miR-612 specifically binds to the 3′-UTR of *TP53* mRNA. Considering the specific regulatory effect of miR-612 on *TP53* expression and the close relationship between *TP53*, HIF-1α, and VEGF, it was speculated that the miR-612-TP53-HIF-1α-VEGF axis regulates the behavior of HBMECs after OM-MSC-EVs.

Transfection of mimics and inhibitor were verified by qRT-PCR, and the results showed that miR-612 expression was significantly upregulated in HBMECs transfected with miR-612 mimic and downregulated in HBMECs transfected with miR-612 inhibitor. Using qRT-PCR and western blotting, it was found that miR-612 overexpression significantly reduced both *TP53* mRNA and protein levels (Fig. 5E, F). The mRNA and protein levels of *TP53* were suppressed by miR-612 inhibitor, further supporting the hypothesis that miR-612 blocked the activation of the *TP53* signaling pathway (Fig. 5E, G). In parallel, transfection of miR-612 mimics into HBMECs resulted in increased mRNA and protein levels of HIF-1α and VEGF (Fig. 5E, F). Conversely, transfection with miR-612 inhibitor resulted in decreased expression of HIF-1α and VEGF (Fig. 5E, G). Taken together, these results demonstrate that HIF-1α and VEGF expression is upregulated by miR-612, and that one miR-612 target, *TP53*, may function as a mediator in the miR-612-HIF-1α-VEGF axis.

**Fig. 6:**
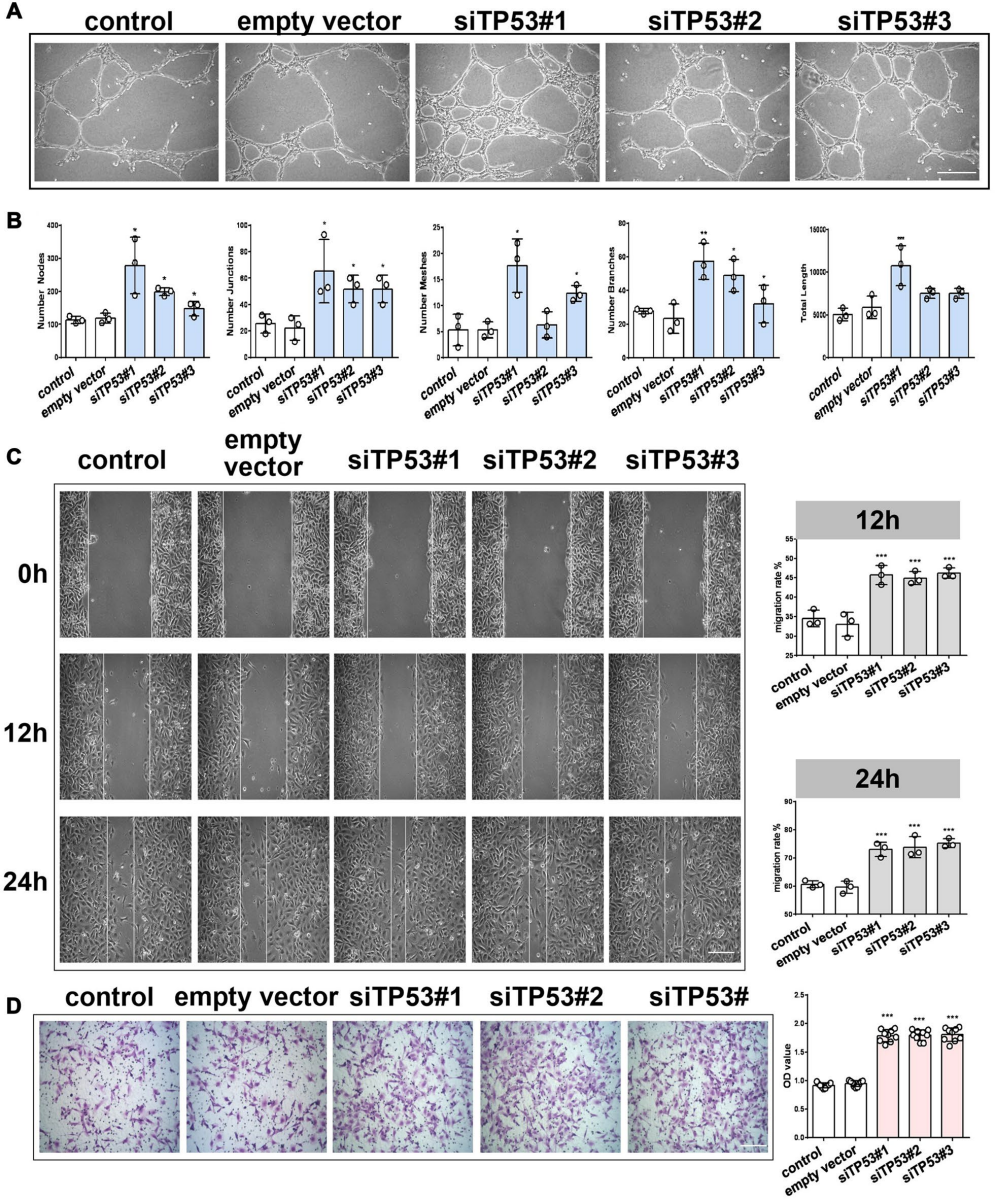
TP53 induced angiogenesis in vitro. **A** Representative images of tubule formation for each treatment group,Scale bar: 200 μm. **B** Quantitative data of tube formation using ImageJ, (n = 3),*P < 0.05, **P < 0.01, ***P < 0.001. **C** Representative images of wound healing assay for each treatment group, and quantitative data of migration rate using ImageJ, (n = 3), ***P < 0.001,Scale bar: 200 μm. **D** Representative images of transwell assay for each treatment group and quantitative data of migrating cells OD value using ImageJ, (n = 9), ***P < 0.001, Scale bar: 100 μm

**Fig. 7:**
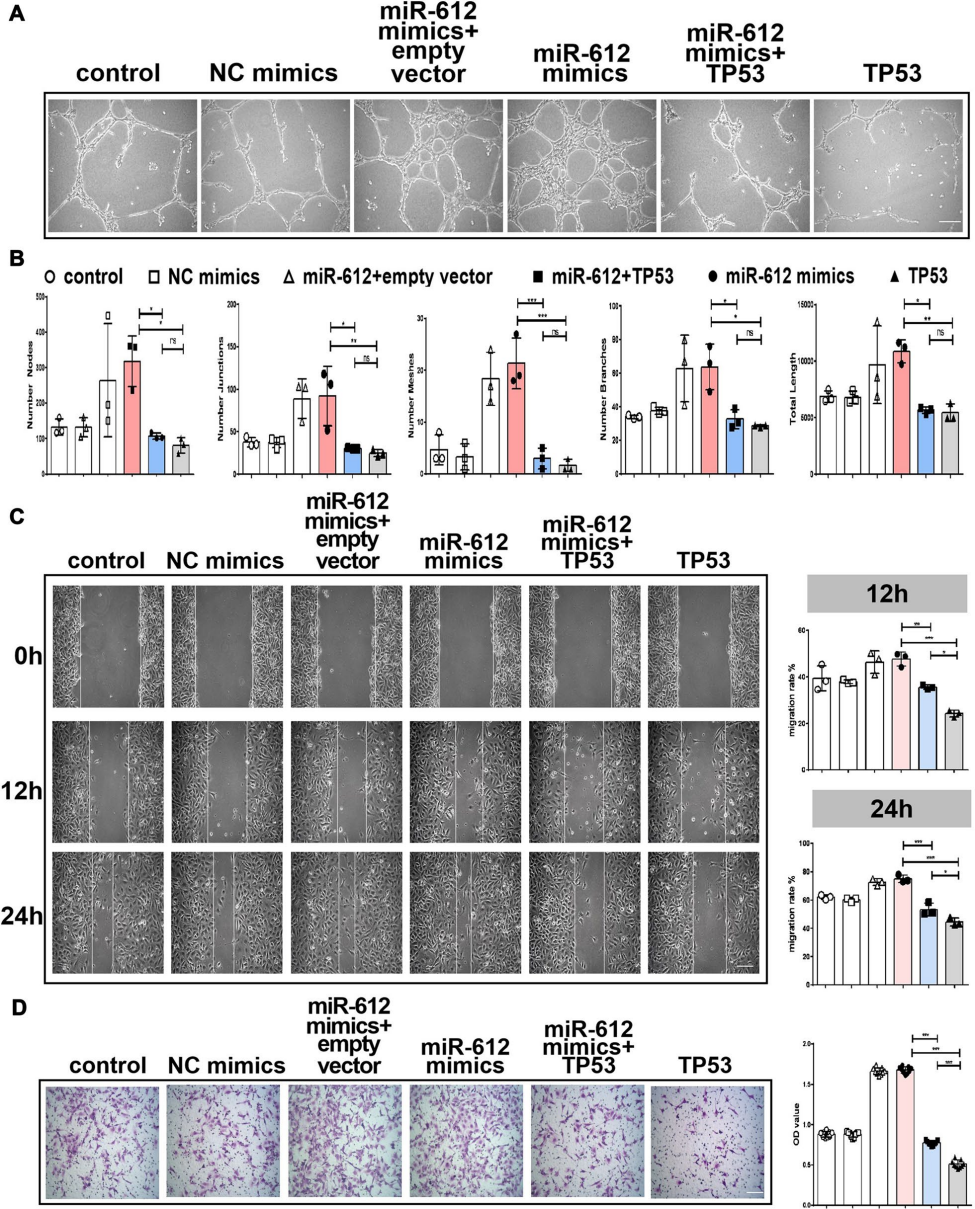
TP53 reduce miR-612-induced angiogenesis in vitro. **A** Representative images of tubule formation for each treatment group, Scale bar: 200 μm. **B** Quantitative data of tube formation using ImageJ, (n = 3), *P < 0.05, **P < 0.01, ***P < 0.001,ns: no significance. **C** Representative images of wound healing assay for each treatment group, and quantitative data of migration rate using ImageJ, (n = 3),*P < 0.05, **P < 0.01, ***P < 0.001, Scale bar: 200 μm. **D** Representative images of transwell assay for each treatment group and quantitative data of migrating cells OD value using ImageJ, (n = 9), ***P < 0.001, Scale bar: 100 μm

Furthermore, the effect of *TP53* on angiogenesis was investigated. HBMECs were transfected with pcDNA3.1-TP53 and *TP53* siRNA (siTP53#1, siTP53#2, and siTP53#3) to upregulate and downregulate *TP53*, respectively. First, the angiogenic activities of HBMECs were assessed after *TP53* interference using specific siRNAs (si*TP53*#1, si*TP53*#2, and si*TP53*#3). Downregulation of *TP53* was verified by qRT-PCR and western blotting (Additional file 1: Fig. S3A, B). As evidenced by the tube formation assay (Fig. 6A, B), scratch wound assay (Fig. 6C), transwell assay (Fig. 6D), and CCK-8 assay (Additional file 1: Fig. S3E), the migration, angiogenic tubule formation, and proliferation of HBMECs were profoundly augmented by si*TP53* #1, respectively, compared with the control siRNA group and empty vector group (Fig. 6A–D). These results indicate that inhibition of *TP53* mediates the pro-angiogenic effects of OM-MSC-EVs on endothelial cells. To assess whether *TP53* mediates the pro-angiogenic activity of miR-612, *TP53* overexpression plasmid was used to upregulate itself in the presence of miR-612 mimics. The efficiency of *TP53* was tested by qRT-PCR and western blot (Additional file 1: Fig. S3C, D). As a result, *TP53* overexpression attenuated the pro-angiogenic activity of miR-612, as determined by the tube formation assay (Fig. 7A, B), scratch wound healing assay (Fig. 7C), transwell assay (Fig. 7D), and CCK-8 assay (Additional file 1: Fig. S3F). Taken together, the in vitro functional assays in HBMECs suggest that controlling *TP53* expression is at least partly responsible for how miR-612 promotes angiogenesis.

### miR-612 is abundant in H-EVs and promotes endothelial cell angiogenesis in vivo

**Fig. 8:**
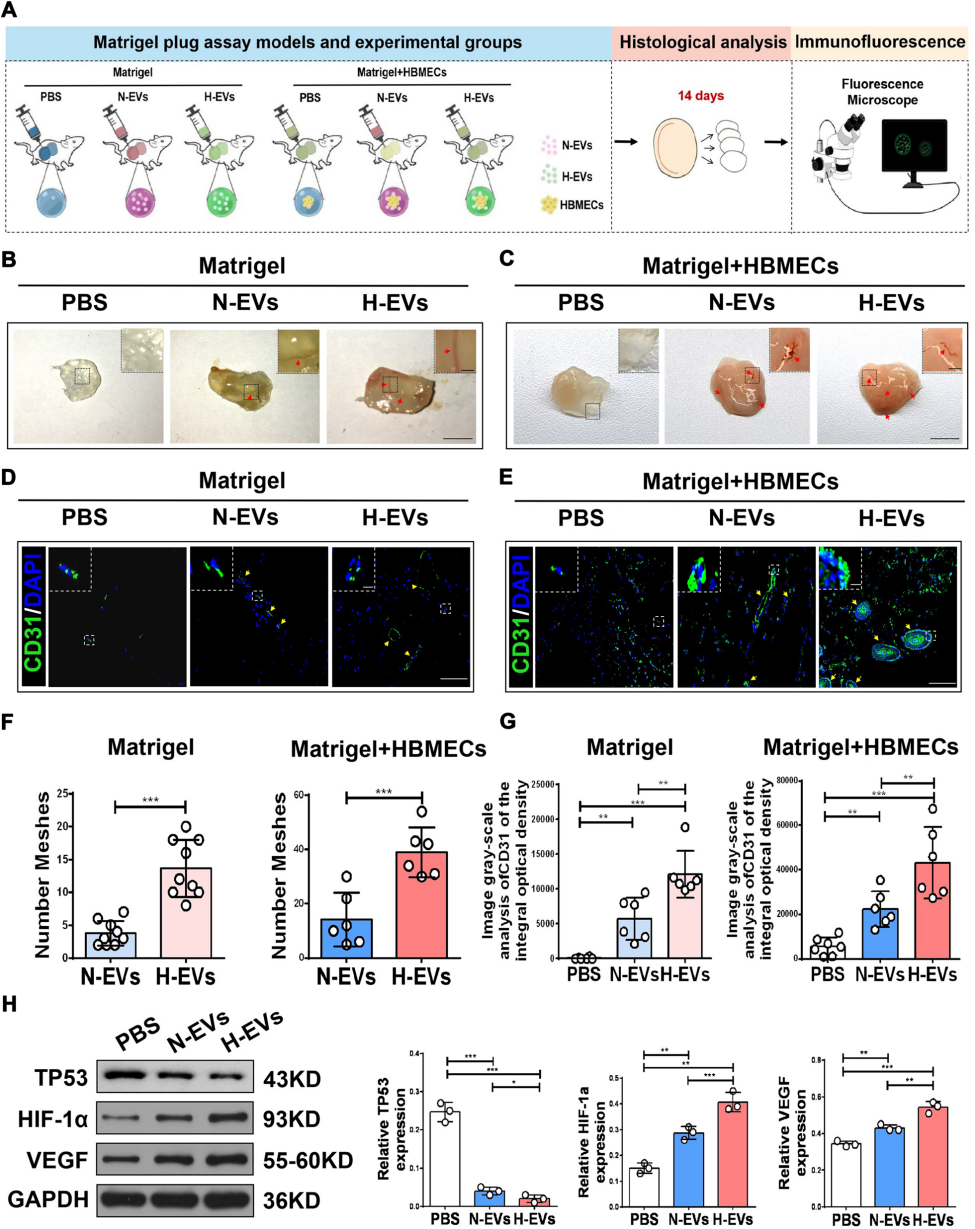
H-EVs promoting endothelial cell angiogenesis in vivo. **A** Schematic representation In vivo experimental design. **B** Macroscopic view of the Matrigel plug (Matrigel only group). The H-EVs treatment group can significantly promote the formation of blood vessels in the Matrigel plugcompared to the N-EVs treatment group, (n = 3), scale bar: 5 mm (for macroscopic images), scale bar: 1 mm (for box images), red arrows indicate blood vessels. **C** Macroscopic view of the Matrigel plug (Matrigel + HBMECs group). H-EVs and N-EVs treatment can promote blood vessel formation in the matrigel, (n = 3), scale bar: 5 mm (for macroscopic images), scale bar: 1 mm (for box images), red arrows indicate blood vessels. **D** The formation of branching capillaries in Matrigel (Matrigel only group), expressing CD31 (green), (n = 3), scale bar: 100 μm (for immunohistochemical images), scale bar: 50 μm (for box images), yellow arrows indicate blood vessels. **E** The formation of branching capillaries in Matrigel (Matrigel + HBMECs group), expressing CD31 (green), (n = 3), scale bar: 100 μm(for immunohistochemical images), scale bar: 50 μm (for box images), yellow arrows indicate blood vessels. **F** The meshse of a plug staining positively with anti-CD31was quantitated using image J (Matrigel only group and Matrigel + HBMECs group), (n = 6), **P < 0.01, ***P < 0.001. **G** The area of a plug staining positively with anti-CD31 was quantitated using image software (Matrigel only group and Matrigel + HBMECs group), (n = 6), **P < 0.01, ***P < 0.001. **H** Western blot analysis of the expression of TP53, HIF-1α, and VEGF in invading cells (Matrigel + HBMECs group) treated with H-EVs and N-EVs,and quantitative data of western blot analysis, (n = 3), *P < 0.05, **P < 0.01, ***P < 0.001

To assess the pro-angiogenic potential of H-EVs in vivo, a Matrigel plug assay was performed in athymic nude mice (Fig. 8A). Test groups included the following: Matrigel only, Matrigel + HBMECs, Matrigel + N-EVs, Matrigel + H-EVs, Matrigel + HBMECs + N-EVs, and Matrigel + HBMECs + H-EVs. These mixtures were subcutaneously injected into mice and the Matrigel plugs were removed after 14 days. Visual examination of the Matrigel plugs showed no new blood vessel formation in the Matrigel only and Matrigel + HBMECs groups, whereas blood (red blood cells shown in red color) and blood vessels were observed in the other four groups (Fig. 8B, C). Matrigel plugs in both H-EV groups had many more vessels than both of the N-EV groups (Fig. 8B, C). Results were validated by immunostaining for the endothelial biomarker CD31 (Fig. 8D, E). Immunostaining quantification for CD31-positive signals and meshes gave similar results (Fig. 8F, G). In the Matrigel + HBMECs + H-EVs group, the numbers of CD31-positive vessels were greater, and the vessel walls were thicker. These results indicated that all OM-MSC-EV groups induced new vessel formation in vivo. Moreover, EVs derived from hypoxic OM-MSCs induced greater vessel formation than those derived from normoxic OM-MSCs. Western blotting analysis showed that the expression of *TP53* was reduced in the Matrigel + HBMECs + N-EVs and Matrigel + HBMECs + H-EVs groups. The decrease of *TP53* in the Matrigel + HBMECs + H-EVs group was greater than that in the Matrigel + HBMECs + N-EVs group (Fig. 8H). However, the expression of HIF-1α and VEGF were increased in both Matrigel + HBMECs + N-EVs and Matrigel + HBMECs + H-EVs groups, the expression of HIF-1α and VEGF in the hypoxic group was higher than that in the normoxic group (Fig. 8H). These results are consistent with what we found in vitro experiments.

**Fig. 9:**
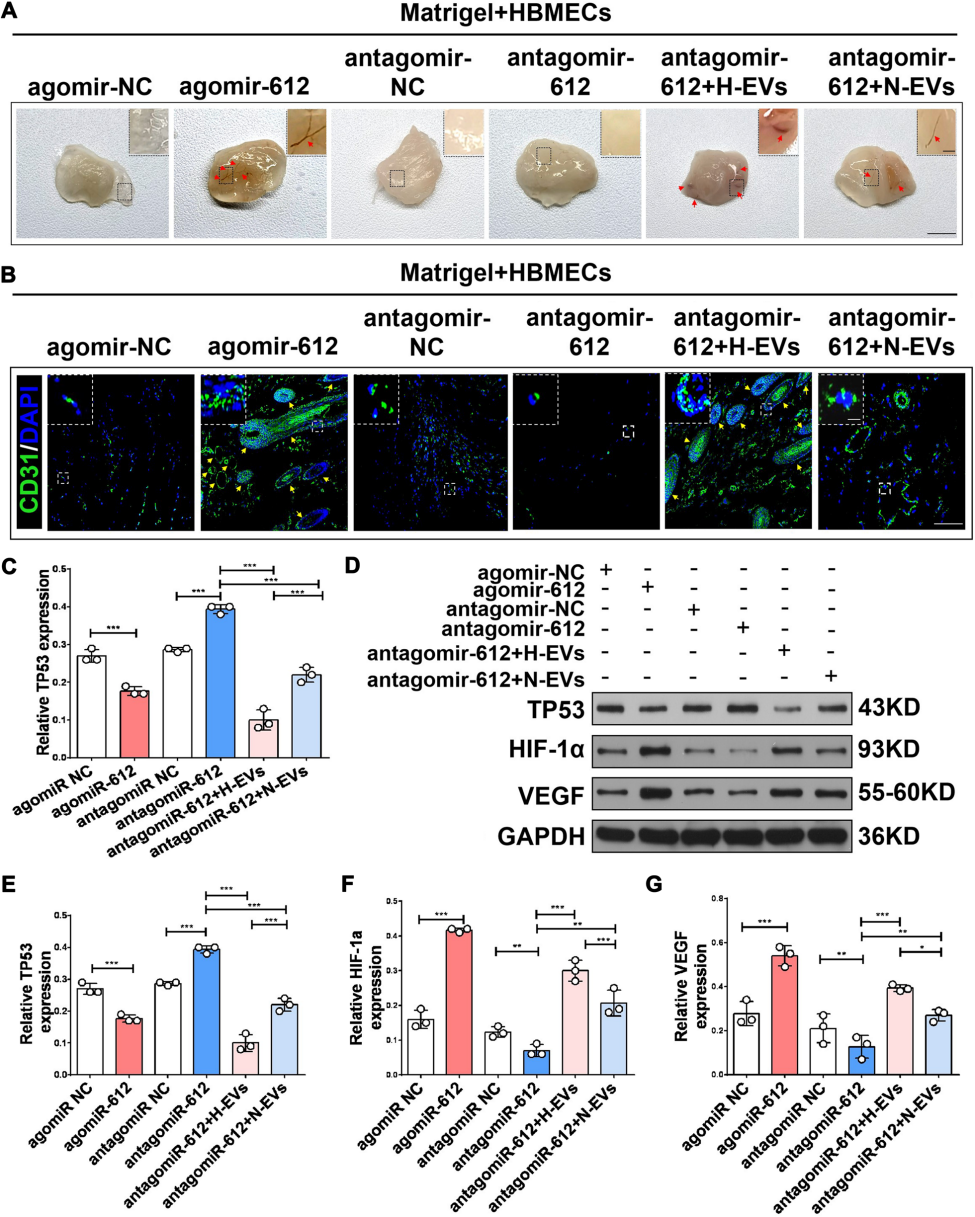
The miR-612 is abundant in H-EVs promoting endothelial cell angiogenesis in vivo. **A** Macroscopic view of the Matrigel plug. The Matrigel plug in the miR-612 agomir group showed much more vessels than other groups. H-EVs, and compared with N-EVs treatment, H-EVs can more significantly reverse the inhibitory effect of antagomir-612 on angiogenesis, (n = 3), scale bar: 5 mm (for macroscopic images), scale bar: 1 mm (for box images), red arrows indicate blood vessels. **B** Immunofluorescence staining observes the morphology and number of blood vessels in Matrigel plugs, (n = 3), scale bar: 100 μm (for immunohistochemical images), scale bar: 50 μm (for box images), yellow arrows indicate blood vessels. **C** The area of a plug staining positively with anti-CD31 was quantitated using image software, (n = 3), *P < 0.05, **P < 0.01, ***P < 0.001. **D**–**G** Western blot analysis of the expression of TP53, HIF-1α, and VEGF in invading cells treated with each treatment group,and quantitative data of western blot analysis, (n = 3), *P < 0.05, **P < 0.01, ***P < 0.001

Thus, H-EV-enriched miR-612 might modulate TP53 signaling in vivo. Six groups, including Matrigel containing HBMECs transfected with an miR-612 agomir negative control (agomir NC group), miR-612 agomir (miR-612 agomir group), miR-612 antagomir negative control (antagomir NC group), miR-612 antagomir (miR-612 antagomir group), miR-612 antagomir + H-EVs (miR-612 antagomir + H-EVs group), or miR-612 antagomir + N-EVs (miR-612 antagomir + N-EVs group) were injected into athymic nude mice. Matrigel was excised after 14 days. The presence of blood vessels was subsequently assessed by immunofluorescence staining for CD31 (green). Consistent with the in vitro data, the number of vessels in the miR-612 agomir group was significantly increased compared with that in the agomir NC group, antagomir NC group, and miR-612 antagomir groups. Matrigel plug in the miR-612 agomir group demonstrated many more vessels than other groups (Fig. 9A). Moreover, the morphology and number of vessels in Matrigel plugs were directly visualized by immunofluorescence staining (Fig. 9B). Quantification of immunostaining for CD31-positive signals gave similar results (Fig. 9C). In addition, miR-612 overexpression by agomir transfection significantly decreased the expression of *TP53* and increased the expression of HIF-1α and VEGF (Fig. 9D–G). Conversely, antagomir-612 significantly attenuated the pro-angiogenic effect of HBMECs (Fig. 9A) as revealed by quantification of CD31-positive signals (Fig. 9B, C). Western blotting analysis indicated that transfection of antagomiR-612 can significantly increase the expression of TP53 and decrease the expression of HIF-1α and VEGF (Fig. 9D–G). Finally, these results were again reversed when the HBMECs transfected with antagomir-612 were incubated with H-EVs and N-EVs (Fig. 9A–G). Taken together, these results demonstrate that H-EV-enriched miR-612 regulates *TP53* signaling in vivo.

## Discussion

The present study demonstrates that OM-MSC-EVs might enhance angiogenesis in vitro and in vivo, especially after hypoxic pretreatment. It was also revealed that OM-MSC-EVs promote HIF-1α-VEGF signaling in HBMECs through the miR-612-TP53-HIF-1α-VEGF axis, therefore suggesting that H-EVs serve as a promising alternative treatment for ischemic disease.

Angiogenesis is a key process in tissue repair after ischemia that involves various cell types, such as endothelial progenitors and inflammatory cells [26]. MSCs have already been shown to promote angiogenesis after ischemia through their differentiation and paracrine signaling activity [27, 28]. Accumulating evidence has suggested that transplanted MSCs promote angiogenesis mainly through paracrine mechanisms, such as EVs, which have been described as the most important effective ingredients that play a significant role in cell-to-cell communication. EVs have been widely used as a natural nanocarrier for the delivery of therapeutic agents into cells due to their advantages in size, structure, stability, and biocompatibility [29–33]. MSCs and their EVs are found in many human organs and tissues, including adipose tissue [34], bone marrow [35], umbilical cord [36], umbilical cord blood [37], placenta [38], and urine [20]. OM-MSCs are localized in the nasal lamina propria and are a novel source of MSCs identified in recent research [10]. A population of OM-MSCs was identified that originate from the olfactory lamina propria, possessing the typical characteristics of stem cells. Due to MSCs' pro-angiogenesis abilities, they have been widely utilized in the treatment of various ischemic diseases. There has already been evidence that OM-MSCs possess several advantages over BM-MSCs. An autologous transplant can be performed using OM-MSCs derived from the nasal lamina propria. In addition, OM-MSCs demonstrated a higher proliferation profile and greater suppressive capacity compared to BM-MSCs [39, 40]. OM-MSCs have been demonstrated to exert protective effects in various disease states, including a Parkinson’s disease [41], global cerebral ischemia [42], cerebral I/R injury [12, 13], hippocampal lesions [10], and autoimmune arthritis [43]. Moreover, it has been reported that OM-MSC-derived EVs ameliorate murine Sjögren’s syndrome by modulating the function of myeloid-derived suppressor cells [40]. However, no study has investigated the angiogenesis-promoting effects of OM-MSC-EVs and H-EVs. To further explore the effect of EVs derived under hypoxic conditions, a series of experiments were conducted to verify that EVs excreted by OM-MSCs under hypoxic conditions have a strong pro-angiogenic effect. Endothelial cells are the major effector cells in tissue repair after ischemia. Their proliferation, migration, and tube formation are essential for angiogenesis. In the present study, the effects of H-EVs on the behavior of endothelial cells (HBMECs) in vitro were evaluated. The results revealed that these nanoparticles can be internalized by HBMECs, and can significantly enhance their proliferation, migration, and angiogenic tubule formation, which confirmed the pro-angiogenic property of H-EVs. In the experiment, we found that H-EVs seemed to be internalized by HBMECs more effectively than N-EVs, although there is no statistical analysis to support this. Additionally, there has been evidence that MSCs derived from other sources can be more efficiently internalized by endothelial cells following hypoxic preconditioning [34, 44]. Hence, we will verify whether H-EVs are internalized more by HBMECs in the future. Further studies have shown that H-EVs increase vascularization of implanted Matrigel plugs in vivo. These results predicted that H-EVs are a positive regulator of angiogenesis. To the best of our knowledge, this is the first study demonstrating the modulation of endothelial cell angiogenesis by miRNA transfected from H-EVs.

OM-MSC-EVs do not only carry diverse sets of proteins [16], but also contain non-coding RNA and DNA, among which miRNAs are of particular interest. The miRNAs are a class of small non-coding RNA molecules 19–25 nucleotides in length that regulate gene expression by binding to the 3'-UTRs of target mRNA [45]. It is generally accepted that miRNAs exert critical effects on cellular processes, such as proliferation, stemness, apoptosis, invasion, and metastasis [46, 47]. However, the effects of miRNAs secreted by human hypoxic OM-MSC-EVs on endothelial cell angiogenesis are poorly understood. Using deep miRNA-seq analysis, 19 miRNAs differentially expressed between H-EVs and N-EVs were identified. The miR-612 is one of the upregulated miRNAs after hypoxia. Indeed, GO and KEGG analyses of miRNA patterns indicated that processes predominant in hypoxia were related to vesicular trafficking and positive regulation of cell communication, as well as pathways related to angiogenesis. Interestingly, miR-612 was involved in the VEGF signaling pathway. Previous research suggested a role for miR-612 in tumorigenesis. miR-612 has been shown to exhibit tumor-suppressing activity in multiple cancers by regulating major tumor-related biological behaviors. However, the role of miR-612 in angiogenesis remains unknown. The current study demonstrated that miR-612 expression was upregulated in human hypoxic OM-MSCs and H-EVs. In addition, miR-612 expression was remarkably enhanced in endothelial cells, indicating that miR-612 can be transferred from OM-MSC-EVs to recipient cells. To confirm the role of miR-612 in this process, EVs were obtained from OM-MSCs that were pretreated with an anti-miR-612 oligonucleotide (OM-MSC-EVs^anti−miR−612^) or with a scrambled construct as the control (OM-MSC-EV^NC^). Functional assays showed that the angiogenic ability, proliferative ability, and motility of HBMECs were decreased in response to the treatment with the OM-MSC-EVs^anti−miR−612^. Thereafter, the miR-612 mimics and inhibitor were used by directly transfecting miRNA into HBMECs. Indeed, miR-612 knockdown in HBMECs partially diminished their pro-angiogenic activity. The results were consistent with those from knocking down miR-612 in OM-MSC-EVs. Moreover, HBMECs were transfected with miR-612 agomir and antagomir for the gain- and loss-of-function investigation. The miR-612 agomir greatly promoted the number of HBMEC-formed tubes and angiogenesis in Matrigel plugs, whereas antagomir-612 significantly attenuated the pro-angiogenic effect. These findings illustrated that miR-612 plays crucial roles in the pro-angiogenic activity of OM-MSC-EVs. However, the effects induced by the specific inhibitor targeting miR-612 were notably reversed by H-EVs and N-EVs in vitro and in vivo. Interestingly, the H-EV-induced angiogenesis in HBMECs was markedly augmented. These findings suggest that miR-612 is one of the critical mediators in H-EV-induced regulation of HBMEC characteristics. Other signaling molecules may also be involved in this process, which warrants a further investigation.

*TP53* is a central component of most cellular stress responses [48]. After activation, *TP53* can positively or negatively regulate the expression of numerous target genes involved in various essential cellular processes, including cell proliferation, survival, and angiogenesis [49, 50]. To further determine the role and molecular mechanism of EV-transfected miR-612 during angiogenesis, computational bioinformatics were used to predict whether *TP53* is a potential target of miR-612. PCR, western blot, and luciferase assays confirmed that miR-612 bound directly to the 3’UTR of *TP53* mRNA and inhibited its translation. Previous studies have found that *TP53* can suppress angiogenesis by transcriptional repression of VEGF expression through regulation of HIF-1α [51, 52]. In accord with these published findings, results of the present study showed that the *TP53* expression levels were significantly decreased, whereas the activities of HIF-1α-VEGF signaling were markedly augmented in HBMECs stimulated by the miR-612-contaning H-EVs and N-EVs. Furthermore, HBMECs were transfected with pcDNA3.1-TP53 and *TP53* siRNA to up-regulate and down-regulate *TP53*, respectively, to investigate its effect on angiogenesis. As a result, *TP53* overexpression attenuated the pro-angiogenic activity of miR-612, indicating that controlling *TP53* expression is at least partly responsible for how miR-612 promotes angiogenesis. Collectively, these findings suggest that *TP53* is a strong mediator in this signaling axis. To the best of our knowledge, the results from both in vitro and in vivo data suggested for the first time that miR-612 likely contributes to the process of angiogenesis. The detailed mechanisms underlying how *TP53* regulates VEGF remain largely unclear. The study of angiogenesis will lead to a better understanding of various physiological and pathological processes, such as vascular disease, wound healing, and tumorigenesis [53]. Although the present study demonstrated a potential role of hypoxic OM-MSC-derived miR-612, further research is required in order to determine the overall importance of miR-612 compared to the wider secretome, as well as the mechanisms behind EV-induced mRNA expression. Further investigation of H-EV-transferred miRNAs might focus on the therapeutic modulation of diseases involving angiogenesis.

There are several limitations of our current study. Firstly, it is possible that our in vivo experiments were not sufficient since we were not able to promote angiogenesis in specific models of ischemic disease. A future study will examine specific ischemic disease models in depth, such as cerebral ischemia and ischemia following intracerebral hemorrhage. In addition, while numerous studies have investigated the effects of MSC-EVs on vascular endothelial cell proliferation by using CCK-8, additional experiments are required to confirm the effects [20, 37]. This will be further illustrated in future models of OM-MSC-EVs for specific ischemic diseases. Secondly, since no studies have reported that miR-612 has pro-angiogenic effects, we used agomir and antagmir to overexpress and knock down miR-612 in target cells. To determine whether miR-612 has an angiogenesis-promoting effect. Future work will use EVs collected from knockdown or over-expressed OM-MSCs to directly interact with in vivo models.

Altogether, the present findings demonstrate that H-EVs markedly enhance angiogenesis. In addition, the miR-612 may play a crucial role in the process of H-EV-dependent regulation of angiogenesis. The study results also suggest that EVs are important mediators of OM-MSC function and can be utilized as a novel therapeutic nano-delivery system for ischemic disease. Therefore, hypoxic preconditioning of MSC-derived EVs represents a novel strategy for the clinical treatment of ischemic diseases with stem cell-derived products.

## Supplementary Information

**Additional file 1: Figure S1. **Characterization of N-OM-MSCs and H-OM-MSCs and EVs uptake assay.** Figure S2. **Hypoxia Leads to Changes in H-EVs and N-EVs miRNA Profiles.** Figure S3. **Evaluation of TP53 transfection efficiency and CCK-8 analysis.

**Additional file 2: Table S1.** Differentially expressed miRNA(|log2FoldChange|> 2, pvalue < 0.05).

## Abbreviations

OM-MSCs: Olfactory mucosa mesenchymal stem cells
EVs: Extracellular vesicles
GO: Gene ontology
KEGG: Kyoto encyclopedia of genes and genomes
miRNAs: MicroRNAs
CCK-8: Cell counting kit-8
qRT-PCR: Quantitative real-time PCR
siRNAs: Small interfering RNA
HIF-1α: Hypoxia-inducible factor 1-alpha
VEGF: Vascular endothelial growth factor
