# Supplementary material for: Extracellular vesicles derived from hypoxia-preconditioned olfactory mucosa mesenchymal stem cells enhance angiogenesis via miR-612
Source: J Nanobiotechnology. 2021 Nov 21;19:380. doi: 10.1186/s12951-021-01126-6 (PMC8607643; doi:10.1186/s12951-021-01126-6)
Supplement: Supplementary file 1 — Additional file 1: Figure S1. Characterization of N-OM-MSCs and H-OM-MSCs and EVs uptake assay. Figure S2. Hypoxia Leads to Changes in H-EVs and N-EVs miRNA Profiles. Figure S3. Evaluation of TP53 transfection efficiency and CCK-8 analysis. [file 12951_2021_1126_MOESM1_ESM.doc]

**Additional file 1**

**Fig.S1: Characterization of N-OM-MSCs and H-OM-MSCs and EVs uptake assay.**

A. Flow cytometric analysis of the fifth passage cells showed that both N-OM-MSCs and H-OM-MSCs express the MSC markers CD90, CD105, CD133, CD146, CD44, and CD73. In addition, cells did not express CD34 and CD45 both hematopoietic cell markers.

B.The fluorescence PKH-67 image of dye-only group (centrifugal supernatant after EVs staining )

**Fig.S2: Hypoxia Leads to Changes in** **H-EVs and N-EVs miRNA Profiles**

A. Identification for categories of small RNA (rRNA, tRNA, snRNA, Cis-reg, other-Rfam-RNA, gene, repeat, known-miRNA and unannotation) from H-EVs and N-EVs.

The percentage of miRNAs in the total RNA isolated from H-EVs and N-EVs, (n = 3), ns: no significance.

B. Venn diagrams showing the distribution of H-EVs and N-EVs.
A. Gene Ontology (GO) analyses.

**Fig.S3: Evaluation of TP53 transfection efficiency and CCK-8 [analysis](../../../../D:/%25E8%25BD%25AF%25E4%25BB%25B6/360%25E6%25B8%25B8%25E8%25A7%2588%25E5%2599%25A8/Youdao/Dict/8.9.9.0/resultui/html/index.html#/javascript:;).**

A, B: Downregulation of *TP53* for each siRNA transfered in HBMECs was verified by qRT-PCR and western blotting.

C, D: The transfection efficiency of *TP53* for each treatment group was tested by qRT-PCR and western blot.

E: CCK-8 assay for each *TP53* siRNA group, (n = 3), ***:*P*<0.001.

F：CCK-8 assay for each treatment group, (n = 3), ***:*P*<0.001.
